# Antibiotic resistance in Vietnam: moving towards a One Health surveillance system

**DOI:** 10.1186/s12889-018-6022-4

**Published:** 2018-09-24

**Authors:** Marion Bordier, Aurelie Binot, Quentin Pauchard, Dien Thi Nguyen, Thanh Ngo Trung, Nicolas Fortané, Flavie Luce Goutard

**Affiliations:** 1 CIRAD, UMR ASTRE, Hanoi, Vietnam; 2 ASTRE, Univ Montpellier, CIRAD, INRA, Montpellier, France; 3 National Institute of Veterinary Research, Hanoi, Vietnam; 4 CIRAD, ASTRE, 34398 Montpellier, France; 5 Vietnam National University of Agriculture, Faculty of Political and Social Sciences, Hanoi, Vietnam; 6 INRA, UMR IRISSO, Université Paris Dauphine, Institut de Recherche interdisciplinaire en sciences sociales, 75116 Paris, France; 7 CIRAD, UMR ASTRE, 10900 Bangkok, Thailand; 8 Faculty of Veterinary Medicine, Kasetsart University, 10900 Bangkok, Thailand

**Keywords:** One health, Surveillance, Antibiotic resistance, Stakeholder analysis

## Abstract

**Background:**

The international community strongly advocates the implementation of multi-sectoral surveillance policies for an effective approach to antibiotic resistance, in line with the One Health concept. To comply with these international recommendations, the Vietnamese government has issued an inter-ministerial surveillance strategy for antibiotic resistance, including an integrated surveillance system. However, one may question the ability and willingness of surveillance stakeholders to implement the collaborations required. To assess the feasibility of operationalising this strategy within the national context, we explored the role of key stakeholders in the strategy, as well as their abilities to comply with it.

**Methods:**

We conducted a qualitative approach based on an iterative stakeholder mapping and analysis, in three distinct steps: (1) a description of the structure of the national surveillance strategy (literature review, key informant interviews); (2) an analysis of the key stakeholders’ positions regarding the strategy (semi-structured interviews); (3) the identification of factors influencing the operationalisation of the collaborative surveillance strategy (comparison of data collected at the first and second steps).

**Results:**

The mapping of the surveillance system, as well as the characterisation of key stakeholders according to organisational and functional attributes, underlined that inter-sectoral surveillance initiatives do exist, but that the organisation of the national surveillance system remains highly silo-oriented.

Based on stakeholder perspectives, we identified seven factors that may influence the implementation of the One Health strategy at national level: governance and operational frameworks, divergence of institutional cultures, level of knowledge, technical capacities, allocation of resources, conflicting commercial interests and influence of international partners.

**Conclusions:**

The study suggests that the operationalisation of the collaborative surveillance strategy requires the full adhesion of stakeholders and the provision of appropriate resources. Based on these findings, we have proposed a guidance framework together with recommendations to move towards a more suitable governance and operational model for One Health surveillance of antibiotic resistance in Vietnam.

To lever and promote successful inter-sectoral collaboration, a participatory “learning by doing” process could be applied to guide, frame and mentor stakeholders through the identification of appropriate levels of collaboration, depending on the expected positive impacts on the value of surveillance.

## Background

Antibiotic resistance (ABR) has been recognised as a global health issue. Resistant bacteria exist in humans, animals, food and the environment, and there are no barriers to the transmission of resistance genes across bacterial species and compartments. Bacteria can be naturally resistant; however, exposure to antibiotics is one of the main drivers for the emergence and spread of resistance genes [1, 2].

Surveillance aims to collect data over time on health events (disease, food contamination, syndrome, etc.) in a specific population to study its evolution in time and space, and thus inform appropriate decisions for the mitigation of related risks [3].

Due to the complex epidemiology of ABR, the international community strongly advocates the implementation of integrated surveillance systems at national, regional and global levels, referring to the One Health concept which promotes collaborative efforts across sectors and disciplines to achieve optimal health for humans, animals and ecosystems [4]. The World Health Organisation (WHO) has issued a Global Action Plan on Antimicrobial resistance, in collaboration with the World Organisation of Animal health (OIE) and the Food and Agriculture Organisation (FAO) [5]. This action plan calls for the development of knowledge around ABR through surveillance and research. A guideline on Integrated Surveillance of Antimicrobial Resistance (AGISAR) has been issued by the WHO advisory group [6]. This guideline provides the basic information required for countries to establish an integrated surveillance of ABR, including antibiotic use (ABU) in humans, food-producing animals and retail food. Several expected outputs of this integrated approach are described in the literature: a better understanding of transmission routes across compartments, the identification of the relative importance of the different reservoirs in the emergence and maintenance of resistance in humans, the study of the correlation between ABU and ABR within and between sectors (namely animal, human and environmental), the assessment of intervention impacts within and between sectors [2, 7].

However, such integrated surveillance systems call for collaboration involving various governmental authorities working at different scales, as well as private stakeholders, that may well have divergent interests, different surveillance objectives and non-standardised methods for data collection. The ways in which surveillance stakeholders appropriate and implement these recommendations are therefore open to question.

In Vietnam, the Government recognises that ABR represents a multi-dimensional risk and a threat to public health, trade, the economy and, more generally, the over-all sustainable development of the country.^1^ While the current health and economic impacts remain unknown, many studies show that the level of resistance to antibiotics is very high in the human and animal sectors [8, 9]. Supported by international organisations and cooperation, and in line with international recommendations, the Vietnamese authorities have developed an inter-ministerial strategy to combat the phenomenon, including an integrated surveillance system for ABR^2^^,^.^3^

To assess the feasibility of operationalising the ABR surveillance strategy in the national context, we explored how the key stakeholders understood and perceived the inter-sectoral approach promoted by national policy, which is strongly framed by international recommendations. We aimed to identify the rationale behind stakeholders’ willingness to adopt the collaborative system, and to identify factors that may impede or enhance the implementation of a One Health surveillance system for ABR in Vietnam. Based on these findings, we have proposed a guidance framework and recommendations to move towards a more suitable governance and operational model for sustainable One Health surveillance of ABR in Vietnam.

## Methods

This study adopted a qualitative approach relying on inductive inference [10, 11] based on stakeholder perspectives. We based our reasoning on an iterative stakeholder mapping and analysis, conducted in three distinct steps detailed below: (1) a description of the structure of the national surveillance strategy in conjunction with international recommendations (based on a literature review and key informant interviews); (2) an analysis of the key stakeholders’ positions regarding the strategy (based on semi-structured interviews); (3) the identification of factors influencing the operationalisation of the collaborative surveillance strategy.

First, we carried out a structural analysis of the ABR surveillance strategy (including ABU). The objective was to (i) describe the international and national framework (regulations, standards) in which the national strategy is anchored, (ii) detail the organisation of the ongoing and future national surveillance system, (iii) analyse the role, mandates and activities of different stakeholders (institution, organisation or profession), as well as their interactions (including collaborations and information flow). This first step aimed at characterising and mapping the current surveillance system of ABR in Vietnam, as well as the organisational and functional attributes of the stakeholders involved.

Data were collected from two sources. First, a literature review was conducted on international recommendations, standards and guidelines, official Vietnamese documents (national action plans, legal instruments, etc.) and reports (scientific, assessment and analytical reports), related to ABR surveillance in all the different sectors. Three informants were then identified as resource persons based on their involvement in the field of ABR or their knowledge about the health system organisation in Vietnam or the antibiotics supply chain; these were interviewed to obtain an overview of key stakeholders and activities in the field of ABR surveillance.

At the end of this first step, the main stakeholders concerned with the issue of ABR were identified and characterised according to twelve organisational and functional attributes, such as professional sector, type of activities, position in the strategy, engagement in the tasks assigned or inter and intra-sectoral interactions. The complete list of these attributes is presented in Table 1.

**Table 1:** Organisational and functional attributes for the characterisation of the structural position of stakeholders involved in the inter-sectorial surveillance strategy of antibiotic resistance in Vietnam

Attribute	Definition	Possible value
Category of stakeholder	Describes the category the stakeholder belongs to	Government authorities, national research institutes, private sector, international partners
Territorial level	Describes the geographical level at which the stakeholder works	Sub-national, national, supra-national
Supervising authority	Describes the institution which has a direct authority over the stakeholder	Prime Minister, Ministry of Health, Ministry of Agriculture, Ministry of Environment, Ministry of Finances, Ministry of industries and trade, Ministry of sciences and technology, provincial people’s committee
Professional sector	Describes the sector in which the stakeholder works	Animal health, husbandry management, animal feed, human health, food processing and distribution, food safety, plant health, wildlife, fisheries, soil and water, antibiotic production/distribution
Stakeholder’s structural position in the surveillance strategy	Describes the role of the stakeholder as defined in official documents framing the surveillance strategy	Operating (stakeholder officially tasked with surveillance activities), influencing (stakeholder officially identified as supporting surveillance activities), absent (stakeholder without any assigned role in the surveillance strategy)
Stakeholder’s activity status regarding tasks assigned	Describes stakeholder’s engagement in undertaking or supporting surveillance activities	Active (stakeholder already engaged), prospective (stakeholder about to be engaged), absent (stakeholder not engaged)
Surveillance component	Describes the surveillance component in which the stakeholder undertakes action	ABR and or ABU in hospitals, ABR and or ABU in community, ABR and or ABU in food-producing animals, ABR and or ABU in companion animals, ABR and or ABU in plant, ABR and or ABU in wildlife, ABR in soil and water, ABR in food
Surveillance activities	Describes at which stage of the surveillance process the stakeholder is involved	Surveillance planning, data collection, data reporting, data sharing, data management, data analysis and interpretation, data communication, data dissemination
Collaborations	Describes at which stage of the surveillance process collaborations occur	Orientation, coordination, planning, data collection, data analysis/interpretation, dissemination, communication
Interacting partner	Describes, for each collaboration, with which partner the stakeholder interacts	Any of the other institutions identified
Collaboration status	Describes the engagement of partners in the collaboration	Planned, ongoing
Type of collaboration	Describes the type of collaboration in place	Technical and financial support, inter-sectoral collaborations

*ABR* Antibiotic resistance, *ABU* Antibiotic usage

Secondly, based on the previous structural mapping, we selected the 21 key stakeholders identified to be “operating^4^” (i.e. stakeholder officially tasked with surveillance activities) or “influencing^4^” (i.e. stakeholder officially identified as supporting surveillance activities). We added 4 “absent^4^” stakeholders (i.e. stakeholder without any assigned role in the surveillance strategy) because of their strong involvement in ABR surveillance activities or the antibiotic business. In total, we invited 39 informants^5^ representative of the 25 selected stakeholders to be interviewed. Among them, 12 “operating”, 8 “influencing” and 5 “absent” informants accepted to participate to the study through individual or collective semi-structured interviews. These 25 informants are identified as key actors of the surveillance system (henceforth referred to as KASS) to explore their abilities to comply with the inter-sectoral surveillance strategy. The interview grid included 5 parts: i) professional background of the informant and description of his/her activities; ii) potential role in the surveillance process and interactions with other stakeholders; iii) knowledge about the Vietnamese strategy to combat ABR and about surveillance activities in the different sectors; iv) identification of potential changes to improve ABR surveillance in Vietnam; v) point of view regarding the international guidance to support countries in implementing One Health surveillance.

We adopted a participatory approach to conduct these interviews. Informants were first asked to map the system in which their ABR surveillance-related activities take place, including interactions with other stakeholders. Investigators then guided them to draw their ‘ideal’ integrated surveillance system, i.e. additional surveillance components to be included, relevant inter-sectoral collaborations, governance model and necessary resources. They could choose for the interview to be conducted either in English or in Vietnamese. All participants had to sign a consent form before the interview began. Participant and institution anonymity was assured.

Each interview was conducted by a team including at least two or three researchers depending on the context and the need for translation. The number of individuals attending the interview ranged from one to six. The average duration of the interviews was 90 min. Initial handwritten notes were first captured in a transcript together with a picture of the system mapped by the participant(s). Then, from each interview, we analysed the participants’ discourse to bring out a set of abilities’ attributes characterising KASS regarding their understanding of the strategy, as well as their perception and level of adhesion to the collaborative approach promoted at the policy level. Based on these attributes, we then described KASS’ abilities to comply with surveillance strategy prescriptions.

In the third step, we analysed how the abilities of stakeholders (step 2) to comply with the surveillance strategy may influence the implementation of the collaborative surveillance system as defined at policy level (step 1). We identified relevant factors that could act as levers for, or barriers to, the operationalisation of inter-sectoral and multi-disciplinary collaborations. Based on this analysis, we have proposed new governance and operational modalities for ABR surveillance in Vietnam that may open the way to a more effective and sustainable One Health surveillance system that considers stakeholder constraints and realities.

## Results

### Structural analysis of the Vietnamese surveillance strategy

#### The Vietnamese strategy to combat antibiotic resistance

The Vietnamese strategy to combat ABR is set out in two main documents. In 2013, the Ministry of Health issued a Global Action Plan, common to the human, animal and environmental health sectors. This action plan provides for surveillance systems to be set up in the human sector, at the hospital and community level (resistance and consumption), in food-producing animals and in plants (use only). In 2017, the Ministry of Agriculture enacted its own action plan that includes a more specific description of how the animal health sector is to handle the tasks assigned by the Global Action Plan. Surveillance of resistance and usage is planned in livestock and aquaculture. In addition, in 2015, a memorandum was signed between the Ministers in charge of Health, Agriculture, Environment and Trade, in the presence of international partners; it underlines the commitment of the Vietnamese government to combatting ABR through an inter-sectoral approach. In 2016, a National Steering Committee was established at a ministerial level to monitor the implementation of the Global Action Plan. It is chaired by the Ministry of Health, assisted by the Ministry of Agriculture, and brings together representatives of the Ministry of the Environment and the Ministry of Trade. Sub-committees have been set up parallel to this to manage the technical implementation of the strategy in the different domains.

#### The organisation of the collaborative surveillance system for ABR in Vietnam

Sectoral surveillance components are now implemented, or are about to be, in the main domains, as advised in international guidance [5, 6]: humans, food-producing animals, food. The Ministry of Health has been tasked to survey two domains (hospitals and community), which are supervised by two separate departments, respectively the department of medical services and the department of preventive medicine. Regarding animals, the department of Animal Health has been officially assigned by the Ministry of Agriculture to take over the surveillance of ABR and ABU in food producing animals.

The Global Action Plan does not plan a surveillance component for the environment. Only the monitoring of antibiotic residues is mentioned.

#### The structural characterisation and mapping of the stakeholders in relation to the national surveillance strategy

**Fig. 1:**
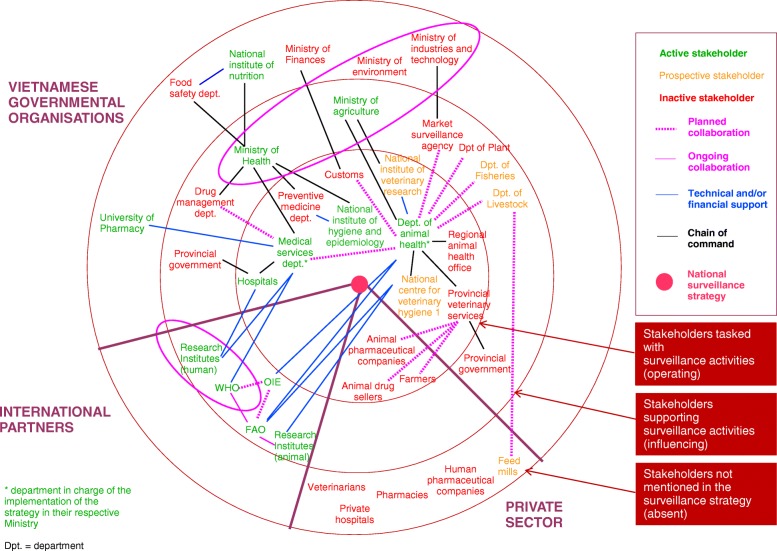
Organisational and functional mapping of the main stakeholders of the ABR surveillance strategy in Vietnam. Surveillance in food-producing animals = Department of Animal Health: management of the surveillance of ABR and ABU in food-producing animals; National center for veterinary hygiene 1: leading laboratory and central unit for ABR, sampling, laboratory testing; Regional animal health office: sampling, laboratory testing; National institute of veterinary research: technical and scientific advice; Provincial veterinary services: collecting data on antibiotic sales and monitoring usage at farm level; Animal pharmaceutical companies, feed mills and drug sellers: reporting sale data; Customs: reporting data on antibiotic importation. Surveillance in humans = Medical services department: management of the surveillance of ABR and ABU in hospitals, central unit for ABR and ABU in hospitals; Hospitals: laboratory testing and reporting data about ABR and ABU; Preventive medicine department: management of the surveillance of ABR and ABU in community; National Institute of hygiene and epidemiology: laboratory testings and central unit (national reference laboratory) for ABR in community. Surveillance in food of animal origin = National institute of nutrition: management and central unit for ABR surveillance, sampling, laboratory testing

We have identified 40 stakeholders directly concerned by the operations of the national surveillance system. 20 belong to governmental authorities (animal health and production, human health, environmental health, plant health, food safety, etc.), six to national research institutes and universities (including hospitals), six to international partners (inter-governmental organisations and foreign research institutes), and 8 to the private sector (feed mills, human and animal pharmaceutical companies, veterinary drug sellers). They intervene in 7 professional sectors: animal health, human health, plant health, environmental health, food safety, animal production, drug manufacturing and distribution. Figure 1 represents a simplified mapping of the stakeholders based on their role in the strategy (operating, influencing, absent^4^) and their current involvement in surveillance activities (active, prospective, inactive^4^). This onion mapping also provides an overview of stakeholder interactions in terms of chain of command or legal supervision, technical and financial support and inter-sectoral collaborations.

Within the governmental agencies, at the operating level, the public health sector is more active than the animal health sector. However, in the preventive medicine sector, in charge of the surveillance at the community level, the involvement of the competent authority remains low and progress in this area is mainly own to the strong commitment of national research institutes. Currently, the cross-sector collaboration remains principally at the policy level, with the constitution of the National Steering Committee. At the operational level, although some inter-sectoral activities are officially stipulated, the operation of the national surveillance system remains highly silo-oriented. Surveillance components are supervised by separate departments with limited communication and collaboration among them, even when under the same ministry. Research institutes are strongly involved in all the different sectors and some, although absent from the surveillance strategy, are involved in certain surveillance activities, either in support of the competent authorities or independently. For instance, the National Institute of Nutrition has initiated the establishment of the surveillance component for food, which was not initially planned in the national strategy, while the National Institute for Hygiene and Epidemiology has developed a project aiming at monitoring resistances at the community level in healthy humans.

International partners are strongly involved in the field of ABR surveillance and provide national authorities with support on a bilateral basis in their respective fields of competencies. They are also closely connected, both in an intra-sectoral and inter-sectoral manner.

The private sector is intended to play an active role in the monitoring of ABU in the animal domain, and certain stakeholders of the antibiotics market chain (pharmaceutical companies and drug sellers) are required to report sales data to governmental bodies. Even though not mentioned in official documents, the feed mills, which are the main providers of antibiotics for animal production should be also solicited by the Department of Livestock to report data on usage. The private sector (healthcare facilities and antibiotic business sector) is absent from the surveillance strategy for ABR and ABU in humans.

### Abilities of the stakeholders involved in the national surveillance strategy

The 25 interviewees belong to 4 professional categories: 7 to governmental authorities (animal health and production, human health, environmental health), 5 to national research institutes (including hospitals), 6 to international partners (inter-governmental organisations and foreign research institutes) and 7 to the private sector (feed mills, human and animal pharmaceutical companies, veterinary drug sellers).

The interviews and participatory mapping sessions enabled us to draw 10 attributes (Table 2) to characterise participants regarding (i) their posture (attitude and position toward the stakes at play in the national strategy), (ii) their technical capital for the implementation of the strategy (capabilities and resources to technically comply with the surveillance strategy), and (iii) their social capital to successfully enable and design inter-institutional relationships. These three attributes are closely linked and influence each other. Both social and technical capitals are notions used in sociology and refer to resources that social stakeholders can rely on and use for their own interest. Bourdieu [12] defined social capital as the entire set of sustainable relationship networks that a social stakeholder (in our context, we are speaking of individuals from institutions, organisations or profession) can mobilise. As a complement to this notion, technical capital is defined as the sum of the stakeholders’ professional network ties and their technical skills, knowledge and resources [13]. Assessing and qualifying the efficacy of such relationship networks and resources require a complex qualitative process that calls upon indicators such as trust, reputation, knowledge and know-how, etc. Thus, we had to accept, within the framework of our study, that the frontier between these notions, used to qualify social stakeholders’ abilities, is loose and porous, as illustrated in Table 2 here below.

**Table 2:** Abilities attributes to describe the posture, technical capital and social capital of the stakeholders

Attributes	Definition
Attributes qualifying the posture	
Legitimacy (relevancy)	Relevance of the stakeholder’s surveillance tasks as defined in the strategy regarding their mandate (governmental bodies) or activities (private sector, research institutes).
Commitment/leadership	Level of willingness of the stakeholder to contribute to the fight against ABR and to act on implementing the surveillance tasks
Confidence	Level of confidence placed by the stakeholder in the success of the surveillance strategy based on (1) capacities of the stakeholders involved, (2) availability of resources, (3) willingness of stakeholders to act against ABR and to collaborate, (4) current situation regarding antibiotics use (including regulation in place)
Attributes qualifying the technical capital	
Capacities	Level of technical capacities of the stakeholder to efficiently undertake surveillance tasks
Resources	Level of resources (material, human and financial) available to the stakeholder to efficiently implement surveillance tasks
Knowledge	Level of understanding of the stakeholder regarding (i) the ABR issue in general and in the context of Vietnam, (ii) the Vietnamese strategy to combat ABR, (iii) surveillance objectives for ABR, (iv) the added value of implementing a collaborative approach compared to a more conventional one.
Attributes qualifying the social capital	
Power	Level of influence of the stakeholder on the implementation of the strategy
Flexibility	Level of freedom of the stakeholder to develop inter-sectoral and inter-disciplinary collaborations or public-private partnerships, especially in the field of ABR surveillance
Willingness to collaborate across sectors and disciplines	Level of interest of the stakeholder to develop collaborations across sectors and disciplines, and especially in the field of ABR surveillance
Trust	Level of trust shown by other stakeholders with regard to this stakeholder based on the latter’s capacities, resources and willingness to collaborate.

Based on informants’ perspectives only, we characterised each of the 4 professional categories according to abilities attributes, and identified trends in their perceptions and position regarding the government’s strategy.

#### Private sector

##### Posture

Private sector stakeholders (both in human and animal activities) clearly understand the public health threat and the stakes at play regarding ABR and demonstrate their interest in joining the combat against ABR. To defend their commercial interests, some are already engaged in a process of public-private partnership with the authorities and antibiotic users to search for alternatives and to promote changing practices. However, they perceive challenges in implementing the national strategy in Vietnam, such as inappropriate legal instruments and regulations, conflicts of interest, under-staffed inspection bodies, etc. They deplore the heavy administrative Vietnamese procedures and the lack of consultation when establishing new regulations.

##### Technical capital

Private sector stakeholder seem to be very well prepared to face the ongoing and upcoming regulatory requirements that come with the implementation of the surveillance strategy. They are already computing data on antibiotic importation, production and sales which are partially reported to the relevant authorities (especially to the customs), on a regular basis or on specific request. “Customs have the best data files in Vietnam”. They argue that they do not foresee any difficulties in reporting more detailed data if requested by the government as they can easily comply with this requirement without any additional resources.

##### Social capital

Human pharmaceutical companies are well organised within a powerful professional organisation on which they can rely to handle concerted discussions with the government when needed. Most of them underline their willingness to actively collaborate with the government and end-users. Other stakeholders involved in the national strategy globally value their experience, the quality of the data they can share and their capacity to assist in the field.

#### National research institutes

##### Posture

National research institutes have been requested to support the different official surveillance components in their respective field of competencies. Globally, national research institutes are highly committed to implementing the tasks assigned; some have also engaged in activities that surpass their mandates and have taken over the role initially assigned to certain competent authorities, considering that the national strategy is currently insufficient or is being implemented too slowly.

Stakeholders working in research institutes are mainly motivated to improve the health situation in Vietnam and are trying to bring scientific evidence to policy-makers to push the surveillance strategy ahead. Nevertheless, research institutes complain about the poor consideration given to the scientific evidence and technical reports for the design of regulatory measures. They feel that even though the regulatory framework is roughly established, authorities are finding it difficult to implement.

Some of them regret the absence of an official risk assessment agency that could provide scientific and technical support to authorities that do not hold the appropriate skills to implement surveillance activities. Independently of the Global Action Plan, some stakeholders involved in the human sector are working to establish a multi-disciplinary and inter-sectoral group to facilitate information sharing and to advise the government.

They also foresee conflicting commercial interests for the public and private stakeholders of the antibiotics market chain; this poses a threat regarding interventions that aim to reduce the use of antibiotics.

##### Technical capital

Researchers usually have a good understanding of the ABR issue and the need to set up a multi-compartment surveillance system, but their expectations and the added value to be derived from a systemic approach remain unclear. The national strategy is usually well known but limited to the sectors in which the stakeholders work.

The stakeholders in charge of ABR in the research institutes are usually well trained (many have an international degree), mainly in microbiology or infectiology, rather than in epidemiology and surveillance. They benefit from substantial technical support from international organisations and bilateral collaborations.

Furthermore, institutes officially tasked with surveillance activities do not receive additional governmental budgets to handle these activities, which are mainly financed with external funds, and they fear that the government will not guarantee funding once these projects have come to a term.

##### Social capital

In each sector, researchers in national institutes are usually very well socially inter-connected with governmental officers, as they have been studying and/or working together for a while.

National research institutes show an interest in surveillance data from other sectors but are rarely engaged in collaborative data sharing processes. When questioned on their form of collaboration, they mention the sharing of results during scientific meetings or through reports and publications.

The fact that international bodies usually develop partnerships with a single institution is considered to induce discrepancies among national research institutes regarding resources, visibility and also access to high-level governmental bodies.

#### International partners

##### Posture

Informants have official mandates to support the development of ABR surveillance or have lengthy experience in working on the topic of ABR. At the inter-governmental level, the role of each organisation is clearly defined within the framework of the OIE-FAO-WHO tripartite.^6^ Most surveillance activities in Vietnam stem from international initiatives, which have usually been endorsed by the government at a high policy level.

They strongly acknowledge ABR as a top health priority and are committed to supporting and assisting the Vietnamese government to address this global challenge. They consider the implementation of the surveillance strategy as a long-term process, and they identify several barriers to its operationalisation, such as the lack of legal instruments to define the role and responsibilities of each stakeholder, or the absence of a regulatory framework to manage data collection on antibiotic consumption in the human and animal sectors. They acknowledge that the national authorities are committed to improving the situation and have noticed that the allocation of national resources is increasing. International partners perceive a strong divergence in institutional cultures between departments, within and between ministries.

In the human health sectors, international activities mainly focus on improving knowledge on the resistance of bacteria and very few initiatives are dedicated to the collection of data on antibiotic consumption.

##### Technical capital

International research partners have a very good technical understanding of the ABR-related issues. They also have extensive knowledge of the Vietnamese strategy to combat ABR when it comes to their professional sector. Nevertheless, they usually lack a systemic approach to the surveillance system and struggle to clearly identify the potential added value of a collaborative approach for surveillance.

In the intergovernmental organisations, ABR focal points are aware of what is going on in the other sectors and have a clear vision of the type of collaboration they foresee as relevant. They consider that the main technical issue for integrated ABR surveillance is to obtain quality data in each sector. Focal points do not usually have specific expertise in surveillance systems and thus contract technical experts to support the authorities in setting up the surveillance activities within the framework of funded projects.

##### Social capital

International partners work closely with the government, which acknowledges how essential their support is. However, their surveillance activities are usually part of a broader project with pre-determined activities and the allocation of specific funds. They therefore often lack the flexibility required to engage in actions that are not directly in line with their project requirements, such as inter-sectoral actions which remain largely neglected. It is also to be noted that, even if the tripartite FAO-OIE-WHO calls for more inter-sectoral collaboration, the country offices or the regional representations do not have a specific budget to achieve this. Projects implemented at country level are usually shaped by international and regional strategies that provide little flexibility to adapt actions to the national context.

International partners work in close collaboration, notably within the tripartite agreement to combat ABR. At the national level, FAO and WHO work within the same office and this is considered to facilitate the sharing of information. In the human sector, international partners meet monthly for a mutual update on their respective surveillance activities.

#### Governmental authorities

##### Posture

Generally speaking, governmental stakeholders have taken action to comply with their assigned tasks regarding surveillance activities. Even though ABR is not considered to be a health crisis in the strict sense of the term, all stakeholders acknowledge the need to join forces in tackling ABR. Progress in this direction is therefore underway, notably thanks to the role played by international partners.

##### Technical capital

The leading departments within the different Ministries have good knowledge on the Vietnamese strategy to combat ABR and on the complex ABR-related issues. They usually attend workshops organised by other sectors, which provides them with insight into recent developments in the field of ABR. However, they have a limited understanding of the added value of setting up a One Health surveillance system in Vietnam. Participants reported that surveillance in hospitals receives much more attention from government and international partners than surveillance at the community level. The major reasons reported are the technical and ethical constraints related to taking samples among healthy humans. Surveillance activities in hospitals are partly funded by city and provincial governments, which may have different resources and allocation priorities. The financial discrepancies between hospitals are considered to impact technical capacities and thus the quality and standardisation of data collection across the country.

Governmental officers also benefit from international capacity building programmes; however, the turnover of key surveillance stakeholders within governmental offices, together with the lack of appropriate human resources management, do not support the maintenance of competencies and knowledge at the institutional level.

##### Social capital

The importance and influence of the different governmental authorities are highly correlated with the support they may obtain from international organisations. At the same time, they have to struggle with hierarchical schemes and pressure due to chains of command and heavy administrative procedures. For instance, when dealing with a cross-cutting issues, they are obliged to set up inter-sectoral committees; certain stakeholders consider that these are too resource and time-consuming and are not adapted to operational surveillance: “They talk a lot but don’t do much”.

Relationships among Ministries and departments are very diverse. For instance, departments belonging to the same or to different ministries may have no history of collaboration or they may have a long-standing history of tension and conflict. Collaboration is mostly limited to the sharing of results across sectors during meetings and workshops.

Regarding relationships with private companies, there remains a need to build trust in the quality of national data, the equality of law enforcement, as well as in the framework of the mandatory consultation process associated to regulation issuing (which is largely considered to be ‘window-dressing’).

### Factors influencing the implementation of the surveillance strategy

Based on the analysis of the abilities of KASS to comply with the strategy, we identified seven factors that may act as barriers or levers to the implementation of the collaborative surveillance strategy.

#### Governance and operational model for the One Health strategy

The Ministry of Health oversees the implementation of the inter-ministerial action plan, while it has no authority over other ministries. Informants considered that this governance model does not promote synergies across ministerial strategies. Furthermore, they mentioned that the National Steering Committee is dedicated to strategic discussions at a high policy level. An operational framework is missing to support technical collaboration. Some sub-committees have been set up to manage the technical implementation of the strategy, but most of these are mainly sectoral and not functional. In Vietnam, it is quite common to set up a National Steering Committee to support inter-sectoral collaboration in the event of a crisis that demands an emergency response, like in the case of highly pathogenic avian influenza (HPAI). It might not be adapted to the fight against ABR, which is not acknowledged as a health crisis by the government, as emphasized by several stakeholders.

The inter-sectoral governance model for ABR is new and does not benefit from a prior inter-sectoral background. Departments which are officially in charge of the implementation of the Global Action Plan, respectively in the animal and human health sectors, have no previous experience of collaboration around a common health issue. Following the avian influenza crisis, some collaborative mechanisms have been established and are framed by regulatory documents that precisely stipulate the roles and responsibilities of the two sectors.^7^ Nevertheless, these existing mechanisms have not been mobilised and adapted to support the collaborative surveillance system for ABR.

#### Co-existence of divergent institutional culture in governmental institutions

Stakeholders reported the co-existence of divergent cultures and competing agendas across governmental departments, belonging to the same or to different ministries. This leads to little sense of mutual understanding and a lack of common goals for surveillance activities. The lack of collaboration between the department in charge of curative medicine and the one in charge of preventive medicine was a recurrent theme. Additionally, participants reported that the department of livestock production and the department of animal health have had divergent perspectives regarding the ban of antibiotics in feed. Participants regularly emphasized that some competent authorities were “easier to collaborate with” than others and were more open to discussions and knowledge sharing. This perception suggests that the culture developed within an institution also influences the willingness of its members to collaborate.

This divergence of culture is mentioned much less frequently between research institutes working in different sectors and supervised by different ministries. They have a long-standing experience of working together. During interviews, they strongly acknowledged the need for multi-disciplinary approaches in the field of ABR that would yield tangible results regarding the development of multi-disciplinary consortiums. This difference of posture between competent authorities and academia may reflect the different types of expectations and motivations they demonstrate. For authorities, surveillance should be primarily sectoral in order to enable them to meet their official mandates, such as managing animal health or public health. For researchers, surveillance yields knowledge which is expected to grow thanks to an increasing range of data types and sources [14].

#### Level of knowledge

The willingness of some stakeholders to collaborate is clearly held back by their lack of perception of the added value to be found in collaborating across sectors in the specific field of ABR surveillance, or of their ability to identify relevant areas of collaboration for surveillance activities. ABR has only recently attracted the attention of policy-makers, and people working in the governmental organisations do not perceive how complex it is or that it is necessary to work together to bring it under control. Most of the stakeholders have limited expertise in epidemiology and surveillance, which is a serious drawback when seeking to set up a collaborative surveillance system. Informants working in research institutes and international organisations deplored the absence of a national risk assessment agency and national reference laboratories in the different sectors; such institutions could efficiently bring scientific and technical support to competent authorities in running the official surveillance system and could advocate a more inter-sectoral approach to surveillance.

In Vietnam, the overuse of antibiotics in the animal sector is considered to be the predominant driver of antibiotic resistance in humans by most of the stakeholders, although the intense pressure exerted by the misuse in people is recognized to play a major role in the development and maintenance of resistances. The blame narrative is not helpful to favour a peaceful time and does not support the development of a mutual respect among sectors.

#### Technical capacities at the sectoral level

Participants often specified that their priority remains the establishment or the strengthening of the surveillance component that they oversee or are involved in. Some of them underlined that effective sectoral components in the different domains are a prerequisite to a meaningful collaborative approach to surveillance. In some domains, stakeholders also indicated that they were not fully confident in the quality of the surveillance data they were currently producing and so preferred not to share it to avoid inappropriate use or controversy during inter-sectoral meetings. Sharing data of poor quality with other sectors and international partners was also perceived as a risk to damage one’s reputation. The recent nativity of the sectoral components is therefore considered as an obstacle to a more collaborative approach, especially in terms of data sharing.

In some governmental departments, the turnover of officers at key positions and the delay in assigning new officers to take over the missions slow down collaboration and the implementation of projects driven by international partners.

#### Governmental financial support

Most surveillance components developed or under development in Vietnam benefit from strong technical and financial support from international partners. Stakeholders deplored the lack of governmental resources dedicated to sectoral surveillance activities and even more dramatically to the implementation of the inter-institutional actions as envisaged in the national strategy. Departments within each ministry are allocated a precise budget, dedicated to activities in their jurisdiction only. There is no targeted funding for collaborative actions, such as the organisation of inter-sectoral work groups or the establishment of a common database, as planned by the strategy.

The lack of governmental resources forces institutions to seek external funding for which they are sometimes in competition with each other; this is perceived as another barrier to collaboration and mutual trust. “We do our things, they do theirs”.

#### Conflicting commercial interests

Antibiotic sales represent a large share of the incomes for all the different stakeholders involved in the antibiotic production and marketing chains, both in the human and animal sectors: pharmacies, veterinary drug stores, pharmaceutical companies and feed mills. Pharmaceutical companies are both private and public, as the State still owns some of the major companies that import, produce and market drugs. In this context, commercial interests were stated by participants to be a potential barrier to the adhesion of some stakeholders, especially those who are requested to report sales data for ABU surveillance in the animal health sector.

#### International partners’ influence

International organisations, both governmental and academic, are very active in Vietnam in the field of ABR surveillance. International partners are also strongly advocating for the implementation of an inter-sectoral approach to surveillance and for more involvement of the private sector and the environmental authority in the surveillance system.

Projects proposed by international partners might be endorsed at high policy level without the consultation of operational stakeholders. This leads to a lack of acceptability that in turn delays their implementation.

Participants deplored that inter-governmental organisations and donors usually support one single department on a bilateral basis. This excludes some stakeholders who feel that they would also be legitimate to receive support in this field. Additionally, this preference dedicated to specific institutions influences, in return, the financial support of the government for one domain to the detriment of another. As a result, some people are frustrated, tension appears across institutions, and this is not in favour of future collaboration.

International partners recognised that they lack flexibility to implement actions at the interface of different sectors. Usually, projects have no dedicated budget for cross-sectoral actions. When the need to support collaborative activities arises during the implementation phase, accountability rules do not allow the allocation of funds for unplanned actions.

## Discussion

### Benefits and biases of the iterative qualitative approach

Based on the analysis of the abilities and willingness of stakeholders to comply with relevant strategies, we identified 7 factors that may act as a barrier for its operationalisation.

It is well recognised that cross-sectoral policies fail to be implemented due to a lack of information on the surrounding political and social environment. The mapping and analysis of stakeholders provides information on key target groups and players who will be impacted by a proposed reform [15]. It helps to predict whether they might support or block the implementation of the latter and thus to propose strategies to promote supportive actions and decrease opposing actions.

The interviews were conducted using a participative approach that enables participants to draw the system they work in and the changes they would like to see in terms of organisation and operations. In such an approach, the active participation of the interviewees favours their empowerment and helps to break down barriers that may exist between respondents and investigators in a more conventional interview. The drawing encourages the participants to structure their answers and the mapping of activities and interactions promotes discussions on their perception of the surveillance system [16]. Furthermore, this method enables the investigator to reformulate information provided more easily and allows the cross-checking of narrative data with the information displayed in the drawing.

We are aware that these results should be interpreted with caution as several factors may have biased them. We assimilated people interviewed with the institution they worked in, but there might be competitive views and attitudes within each institution. Translation might have also introduced some misunderstanding. We were not able to interview some key operating stakeholders, who did not agree to meet us. Finally, the surveillance strategy is very dynamic in Vietnam with a lot of initiatives ongoing, and data collected might be rapidly outdated. However, we believe that the participatory approach implemented in our study has positively impacted the quality of the data collected and alleviated some of these biases.

### Challenges of perceiving added value in the collaborative approach for ABR surveillance

Although ABR has been recognised as a global concern that mandates global coordination [17], it can be considered as a new topic for both academic and governmental stakeholders. Unlike other health hazards with well-investigated epidemiological links between the human, animal and environmental domains and a clear need for collaborative surveillance, such as the West Nile virus [18, 19], appropriate collaboration mechanisms are more difficult to identify in the case of ABR surveillance [2]. Furthermore, in Vietnam, the key governmental stakeholders are not the same as those involved in the fight against zoonotic diseases who have a long standing experience of collaboration, dating back to the emergence of HPAI and who benefit from a legal framework that precisely defines collaboration modalities^7^. The governmental officers engaged in the fight against ABR must therefore be made aware of the necessity of working beyond their institutional boundaries, when it comes to health issues, under the shared responsibility of several professional sectors.

Despite the alarming figures available on the costs and deaths incurred by resistant bacteria [20, 21], the immediate and visible impacts of ABR on health and economics, as compared to other hazards such as HPAI or cancer, are not always clear to stakeholders. As such, it is not a health issue that induces panic and fear in civil society. According to Jerolmack [22], this may act as a barrier to collaboration across sectors, as human and animal health agencies are more successful in aligning their priorities and actions when facing severe disease events. As has also been highlighted for other One Health topics, establishing inter-sectoral collaboration in “non-crisis periods” is really challenging [23].

### Necessity to tailor the collaborative surveillance system to the objective and context

This study underlined that the sectoral surveillance components are not yet well established in Vietnam. Each sector concentrates its efforts on developing or strengthening its own surveillance activities. Operational and efficient sectoral surveillance components are certainly needed to further establish a One Health surveillance system [2]. Nevertheless, inter-sectoral and inter-disciplinary collaboration should not be neglected at this early stage of the surveillance development as this would promote the understanding of ABR as being a “hybrid” issue and help in the development of common goals for its surveillance. To the contrary, the fragmentation of the problematic across different institutions with different organisational cultures would result in an institutional silo-oriented response, shaped by inter-sectoral conflicts and tension over jurisdictions, resources and reputation. Consequently, institutions would struggle to build inter-sectoral and inter-disciplinary bridges when the need to collaborate arises in the future [22]. An inter-sectoral framework should be defined at this early stage of the implementation of the national ABR surveillance system. It could support and guide the stakeholders towards increasing levels of cross-sectoral collaboration, as and when the sectoral surveillance components improve and the collaborative surveillance objectives evolve.

The complex epidemiology of ABR undeniably calls for a global approach to surveillance in line with the One Health concept [24]. Nevertheless, and especially in the case of ABR, One Health surveillance should not be systematically assimilated with integrated surveillance, which suggests the unification of all possible surveillance components within a single and global system (Bordier M, Uea-Anuwong T, Binot A, Hendrikx P, Goutard FL: Characteristics of one health surveillance systems: a systematic literature review. Prev Vet Med., unpublished). ABR surveillance is characterised by the co-existence of several sectoral surveillance components collecting different types of data with various methods and to answer specific sectoral objective(s). Bodies acting and inter-acting in these surveillance components demonstrate different priorities, expectations and constraints. A sustainable and relevant One Health surveillance system thus requires the articulation of the different sectoral components together around a common surveillance objective, defined in a concerted manner with the different stakeholders, rather than their fusion into a single system [14]. Appropriate collaboration modalities must then be established depending on this objective, while taking into account the implementation context (Bordier M, Uea-Anuwong T, Binot A, Hendrikx P, Goutard FL: Characteristics of one health surveillance systems: a systematic literature review. Prev Vet Med., unpublished). As a matter of fact, collaboration leading to the harmonisation and combination of data from different sources would undoubtedly improve knowledge on transmission routes and risk factors related to ABR [2]. However, this should not be done at the expense of the surveillance objective and purpose in each respective sector where priority remains at this stage the reduction of antibiotic consumption and resistance spread in domains under their jurisdiction.

Within the One Health tripartite partnership, international organisations could support countries in establishing appropriate governance models and in identifying the necessary technical collaborations across sectors, depending on their national surveillance context [25].

### Guidance framework to support stakeholders in the operationalisation of One Health surveillance

The study suggests that a collaborative surveillance system for ABR in Vietnam can only be operationalised in a sustainable manner if the full adhesion of the KASS is obtained and if appropriate financial, human and material resources are available.

The willingness of KASS to engage in this collaborative strategy relies on two social conditions: firstly, there must be mutual understanding and trust across institutions and sectors; secondly, stakeholders should perceive the added-value of working beyond their sectoral and disciplinary boundaries, without fearing a loss of their autonomy and leadership within their jurisdiction. Collaboration is resource-consuming both in terms of human and financial resources, and working with people from different cultures and areas of expertise cannot be achieved without stepping out one’s comfort zone [22]. As a result, stakeholders need to see the benefits of linking up to each other to compensate for the cost of their collaborative efforts. In Vietnam, the establishment of a scientific and technical inter-sectoral platform could help to remove organisational barriers and create a climate of trust, by providing a place for exchange and discussions. Along with its networking function, this platform could also play the role of a national risk assessment agency, in charge of supporting the authorities in the operating of the surveillance system: identification of appropriate modalities for collaboration, provision of scientific evidence and technical assistance to the government for the conception of surveillance protocols and for the inter-sectoral interpretation of the data, conducting of technical and economic evaluations of the surveillance system.

The translation of stakeholder adhesion into sustainable collaborative actions requires an appropriate framework for the governance and the operation of the surveillance system. Adequate governance modalities are needed to define the collaborative strategy and to provide necessary guidance and resources for its implementation. In Vietnam, the National Steering Committee should be chaired at the Prime Ministry level to ease the implementation of the global action plan and to ensure synergies across ministerial strategies. A regulatory framework should be defined accordingly to clearly state the role and responsibilities for all stakeholders (authorities, national reference laboratories, national research institutes, private sector) in the implementation of the strategy. Financial mechanisms should be established and approved at a high policy level to ensure the allocation of appropriate resources to surveillance activities, including those requiring inter-sectoral collaboration.

**Fig. 2:**
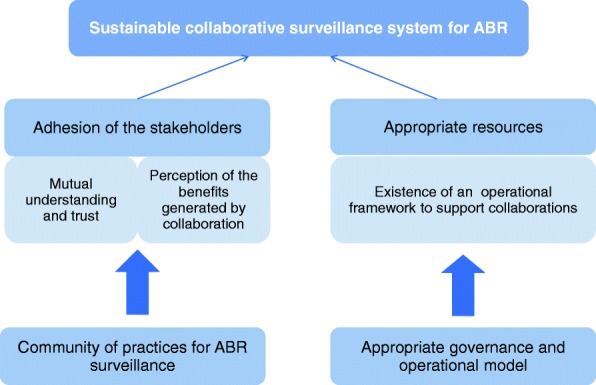
A guidance framework to support the operationalisation of One Health surveillance of ABR in Vietnam

Based on the situation analysis, Fig. 2 proposes a guidance framework to support the operationalisation of sustainable One Health surveillance for ABR in Vietnam. Some of the barriers to collaborations specifically identified through this study are not context dependant as they are also described for other hazards and in different socio-economic setting [26]. This guidance framework could also serve as guidance for the implementation of other surveillance systems requiring collaboration across sectors and disciplines. However, the study has highlighted the benefits of conducting a stakeholder mapping and analysis using an iterative approach, to capture the contextual factors as well as stakeholder perceptions that may influence collaboration in order to identify relevant recommendations to favour the implementation of a One Health surveillance system.

## Conclusions

Adhesion of key social stakeholders to an integrated surveillance system for ABR, as promoted by international organisations, appears to be challenging in the Vietnamese context. To lever and promote successful inter-sectoral collaboration, a participatory “learning by doing” process could be applied to guide, frame and mentor stakeholders through the identification of the appropriate level of collaboration depending on the expected positive impacts on the surveillance value. Such an approach should be designed within an adequate methodological and conceptual framework, as proposed in participatory modelling for instance [27]. This method has been designed for collective decision-making, research and institutional coordination. The main idea is that participatory modelling, by rendering explicit the biological processes as well as stakeholders’ strategies and social relationships, can be used by stakeholders themselves to deal with their own problems and to identify mutually accepted solutions that can lead to collective action. In the context of ABR surveillance in Vietnam, this would probably help to improve trust among stakeholders and their understanding of the benefits to be gained from an inter-sectoral approach, through the concerted definition of practical collaboration modalities and mechanisms, accepted and endorsed by all and one. This approach would progressively lead to the development of an inter-sectoral and multi-disciplinary community of practices, which would support the implementation and operation of a consensual collaborative surveillance system for ABR in Vietnam. The establishment of a scientific and technical inter-sectoral platform could provide an appropriate frame to implement this mentoring approach.

Stakeholder mapping and analysis, followed by the participatory modelling process, would appear to be a promising approach through which to engage stakeholders with different backgrounds and expectations in a collaborative surveillance system. The first tool provides an exhaustive overview of the relevant stakeholders as well as the barriers that may impede their adhesion to collaboration. Based on this result, and using participatory workshops, different scenarios can be co-designed with the stakeholders to seek collective solutions to raise these barriers and to move towards a One Health surveillance system with a well-balanced objective and an acceptable level of integration, that comes as close as possible to meeting expectations.

## Abbreviations

ABR: Antibiotic resistance
ABU: Antibiotic use
AGISAR: A guideline on Integrated Surveillance of Antimicrobial Resistance
FAO: Food and Agriculture Organisation
HPAI: Highly pathogenic avian influenza
KASS: Key actors of the surveillance system
OIE: World Organisation for Animal health
WHO: World Health Organisation

## Footnotes

1 Aide Memoire – Multi-stakeholder engagement to combat Antimicrobial Resistance in Viet Nam - Hanoi, June 24th 2015.

2 Ministry of Health – National Action Plan on combatting drug resistance in the period 2013–2020.

3 Ministry of Agriculture and rural development – National Action Plan for management of antibiotic resistance in livestock production and aquaculture – Decision n°2625/QD-BNN-TY date June 21st 2017.

4 Possible value of the variable “Stakeholder’s structural position in the surveillance strategy” in Table 1

5 Several possible informants for one type of stakeholder.

6 http://www.who.int/foodsafety/areas_work/antimicrobial-resistance/tripartite/en/ (Accessed 26 June 2018).

7 Circular No. 16/2013/TTLT-BYT-BNN&PTNT: “Guidelines for coordinated prevention and control of zoonotic diseases”. Ministry of Agriculture and Rural Development, Ministry of Health.
